# GrainScan: a low cost, fast method for grain size and colour measurements

**DOI:** 10.1186/1746-4811-10-23

**Published:** 2014-07-08

**Authors:** Alex P Whan, Alison B Smith, Colin R Cavanagh, Jean-Philippe F Ral, Lindsay M Shaw, Crispin A Howitt, Leanne Bischof

**Affiliations:** 1 CSIRO Plant Industry, GPO Box 1600, Canberra ACT 2601, Australia; 2 National Institute for Applied Statistics and Research Australia, Univeristy of Wollongong Wollongong NSW 2522, Australia; 3 CSIRO Computational Informatics, North Ryde NSW 2113, Australia

**Keywords:** Wheat, Brachypodium distachyon, Seed size, Seed colour, Image analysis

## Abstract

**Background:**

Measuring grain characteristics is an integral component of cereal breeding and research into genetic control of seed development. Measures such as thousand grain weight are fast, but do not give an indication of variation within a sample. Other methods exist for detailed analysis of grain size, but are generally costly and very low throughput. Grain colour analysis is generally difficult to perform with accuracy, and existing methods are expensive and involved.

**Results:**

We have developed a software method to measure grain size and colour from images captured with consumer level flatbed scanners, in a robust, standardised way. The accuracy and precision of the method have been demonstrated through screening wheat and *Brachypodium distachyon* populations for variation in size and colour.

**Conclusion:**

By using GrainScan, cheap and fast measurement of grain colour and size will enable plant research programs to gain deeper understanding of material, where limited or no information is currently available.

## Introduction

Measurement of seed characteristics is a vital aspect of cereal research. Grain size represents one of the major components of yield, it contributes to seedling vigour [1,2], and larger grains may lead to an increase in milling yield [3-5]. Seed colour is also important for breeding of cereal varieties because it affects the quality and appeal of processed grain, and is also associated with dormancy in multiple species [6,7].

### Grain size

Grain (or seed) size is an important component of both basic plant research, since seed formation and development is a fundamental aspect of plant reproduction, and cereal breeding, as a component of yield and vigour. Existing methods of determining seed size tend to either favor speed of measurement while sacrificing resolution, or are so involved that high throughput measurement is challenging. In the context of cereal breeding, seed weight is an important trait related to seed size, and therefore measuring the weight of a standard number or volume of seeds is practical and informative. Measures such as thousand-grain weight or hectolitre weight are commonly used since they are fast, and not prone to error. However, they give no measure of variation within a sample. Detailed measurement of seed shape characteristics such as length and width traditionally depends on laborious techniques such as manual measurement of individual seeds [8]. The single kernel characterization system (SKCS, [9]) is a relatively low throughput, destructive technique that measures hardness as well as seed size. Systems such as SeedCount (Next Instruments, NSW, Australia) utilize image analysis to give measures of size for individual seeds within a sample, allowing for a detailed understanding of variation, as well as an accurate estimation of the sample mean. However the time required for sample preparation especially for large numbers of samples (SeedCount samples need to be placed in wells in a sample tray), along with the initial cost of such systems can be prohibitive (~ $AUD15000).

### Grain colour

The association between red seed colour and increased dormancy has been recognized in wheat for over a century. Nilsson-Ehle [10], cited in [11] suggested that three genes were controlling red pigmentation in wheat, and subsequently three homoeologous loci have been mapped to the long arm of chromosome group 3 [12] encoding a Myb-type transcription factor having pleiotropic effects on both dormancy and expression of genes in the flavonoid biosynthesis pathway [13]. With increased copy number of red genes (3A, 3B, 3D) there is an additive effect on increasing dormancy in wheat, however other genetic loci such as those on 4AL and 3AS have been found to explain a greater percentage of the genetic variation [14]. White wheat may be more desirable because of increased milling efficiency and consumer preferences for some end products, such as Udon noodles [15].

No simple methods for measuring seed colour (other than human estimation) are available. Colour estimation is generally performed on a modal scale by eye, resulting in loss of colour gradation information (inability to classify gene number). Unless the colour difference is stark, there is a high likelihood of inconsistent estimation [16]. For classification of wheat as genetically either red or white, seeds can be soaked in NaOH to increase the contrast between the two [17], however this is relatively low throughput, and does not take into account further colour variation due to environmental or other genetic factors.

Accurate, widely interpretable measurement of colour is technically challenging, and a field unfamiliar to many biologists. Because perception of colour is affected by the environment in which it is observed, standardised measurement is critical. Such a requirement generally involves somewhat laborious sample preparation and high cost analytical equipment. Chroma meters are standard tools for accurate colour determination in many industries, and can be applied to cereal products along the processing chain, including grain, flour, dough and the final processed product. For standardised, comparable colour measurements, chroma meters measure in the CIELAB colour space, a device independent colour space which includes all perceivable colours. CIELAB is made up of three channels: L*, which ranges from 0 to 100 and represents the lightness of the colour; a*, negative or positive values of which represent green or magenta, respectively; and b*, representing blue (negative) or yellow (positive). These channels can then be used individually to quantify specific colour attributes, which may be linked to biological factors [18]. While the measurements given by chroma meters are highly controlled and standardised, when applied to grain, there are several drawbacks. Because of the small area that is measured, only a limited number of grains are visible by the observer, and a single average value is reported. This, therefore, provides no information regarding variation within a sample of grain. An alternative method is the SeedCount system, which also provides colour information based on the CIELAB colour space, as well as other grain characteristics such as size and disease state.

There is increasing use of image analysis in plant science and agriculture, especially in the field of phenomics [19,20]. While demonstrating great potential in accelerating detailed plant measurements, many of the available methods depend on very costly infrastructure, limiting widespread adoption. Developments in the availability of image analysis for plant measurement applications have made low cost alternatives available, including: RootScan, which analyses root cross sections [21]; Tomato Analyzer, which measures a range of features including shape and disease state in tomatoes and other fruits [22]; and the web application PhenoPhyte, which allows users to quantify leaf area and herbivory from above ground plant images [23]. ImageJ is general purpose image analysis software that is freely available [24], and has been used to analyse seed shape and size parameters in a range of plant species including wheat, rice and *Arabidopsis*[25-28]. SmartGrain [29] is another image analysis system that is free to use, and is also based on images captured by consumer level flatbed scanners to extract seed characteristics. SmartGrain builds ellipses on identified grains to establish seed area, perimeter, width and length, but does not measure colour information. Seed shape can also be analysed with the software SHAPE [30], which produces elliptic Fourier descriptors of 2- and 3-dimensional characteristics from photographs of vertically and horizontally oriented seed, which has the advantage of potentially identifying different loci affecting seed shape, but due to the nature of the image capture, requires manual handling and preparation of individual seeds [31].

Here, we present GrainScan [32], a low cost, high-throughput method of robust image capture and analysis for measurement of cereal grain size and colour. GrainScan utilizes reflected light to accurately capture colour information described in a device independent colour space (CIELAB), allowing comparison of colour data between scanning devices.

## Results and discussion

To test the accuracy of GrainScan, wheat seeds from a diverse mapping population were measured with GrainScan, SmartGrain and Seedcount. These comparisons were used because SmartGrain and SeedCount are specifically designed for grain analysis, and each includes components that provide similar functionality to elements of GrainScan.

### Size traits

**Figure 1:**
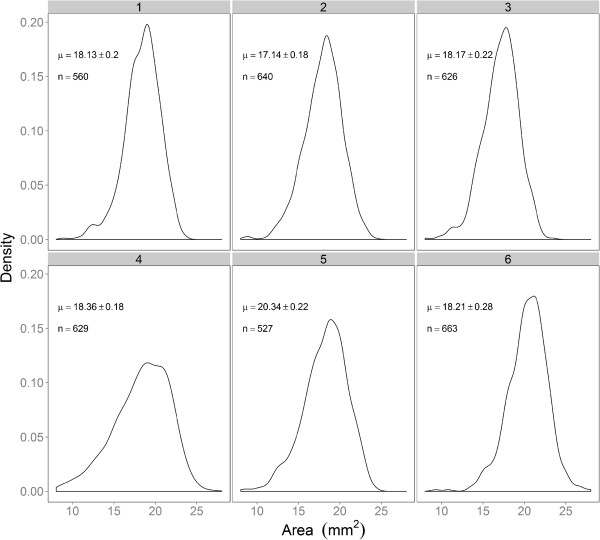
**Density distributions of grain area for six randomly chosen samples of wheat grain.** The mean and confidence interval, along with the number of seeds included in each scan is noted on each panel.

The distribution of size traits measured by GrainScan for individual images could be reasonably approximated by a Guassian distribution (Figure 1). Because of the number of seeds measured in each scan, there was a high level of confidence in the mean trait value for each image.

#### Comparison of screening methods

**Figure 2:**
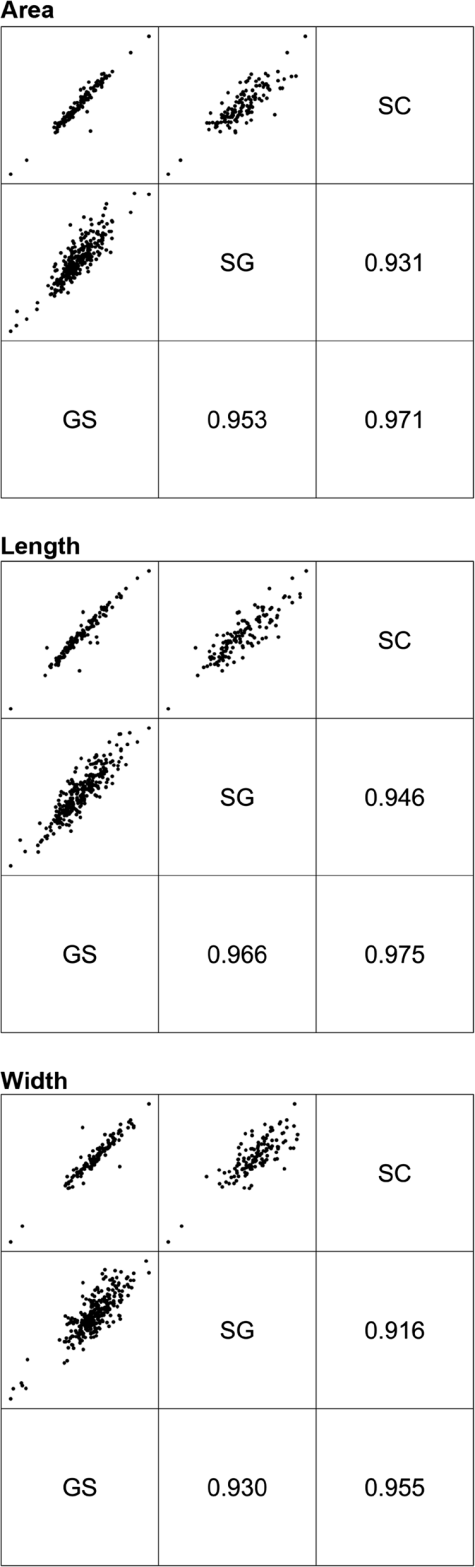
**Correleation of BLUPs for size traits.** Pairwise plot of BLUPs of packet effects (above the diagonal) and REML estimates of correlations between packet effects (below the diagonal) for size traits from GrainScan, SmartGrain and SeedCount. Method labels are on the diagonal: SC (SeedCount), SG (SmartGrain) and GS (GrainScan).

Summary data for each size trait as measured by GrainScan, SmartGrain and SeedCount is shown in Table 1. Mean values and ranges for size traits across the population were similar between methods. The REML estimates of the correlations between the packet effects for different methods are shown in Figure 2. Each correlation gives a measure of the agreement in the ranking of effects between methods. In the context of a breeding program this measure would relate to the similarity between methods in terms of genotype rankings and thence selection. A correlation near +1 suggests identical rankings for the two methods; a correlation near -1 suggests a complete reversal of rankings and a correlation near 0 suggests very little relationship between the rankings. Figure 2 shows that GrainScan correlates highly with both methods for all size traits, but most strongly with SeedCount. The strength of the correlations is also reflected in the pairwise plots of the packet effect BLUPs in Figure 2.

**Table 1:** Summary statistics (minimum, mean and maximum) of raw packet means for each trait and method

	**GrainScan**	**SmartGrain**	**SeedCount**
Area-min	11.68	10.22	10.00
Area-mean	17.99	15.96	16.07
Area-max	24.52	21.34	22.05
Length-min	5.40	5.25	5.36
Length-mean	6.71	6.51	6.71
Length-max	7.99	7.70	7.94
Width-min	2.65	2.47	2.58
Width-mean	3.41	3.24	3.39
Width-max	3.91	3.74	3.88

Seed area is measured in mm^2^, length and width are in mm.

The average accuracy (correlation between true and predicted packet effects, Table 2) for GrainScan was very high (0.981 – 0.996) and similar to SeedCount (0.991 – 0.994) for both replicated and unreplicated packets, while the average accuracy for trait measurements from SmartGrain was lower (0.871 – 0.947).

**Table 2:** Average accuracies for each size trait for each method

	**Unreplicated packets**	**Replicated packets**	**Trait**
GrainScan	0.993	0.996	
SmartGrain	0.900	0.945	
SeedCount	0.992	0.994	Area
GrainScan	0.981	0.990	
SmartGrain	0.903	0.947	
SeedCount	0.994	0.995	Length
GrainScan	0.990	0.994	
SmartGrain	0.871	0.928	
SeedCount	0.991	0.994	Width

Averages are computed separately for unreplicated and replicated packets.

Measurements took approximately twice as long using SeedCount compared to scanning for analysis by GrainScan or SmartGrain (210 seconds and 101 seconds, respectively). This time only considered the image capture, which for SeedCount included image processing time, while for the other methods, image processing was done as a batch after all images were captured. However, the difference in time was mainly due to the time taken to lay out seeds as required in the sample tray for SeedCount, as opposed to scattering in the glass tray for the flatbed scanning. Because wheat grains are rounded, when they are scattered on the glass, they can roll into different orientations. GrainScan provides a facility to detect grain creases (described below), which can be used to filter out data from grains that are not oriented crease down. In our comparison of methods we have used measurements from all visible seeds, since it represents the complete GrainScan output.

### Colour traits

#### GrainScan colour determination

**Figure 3:**
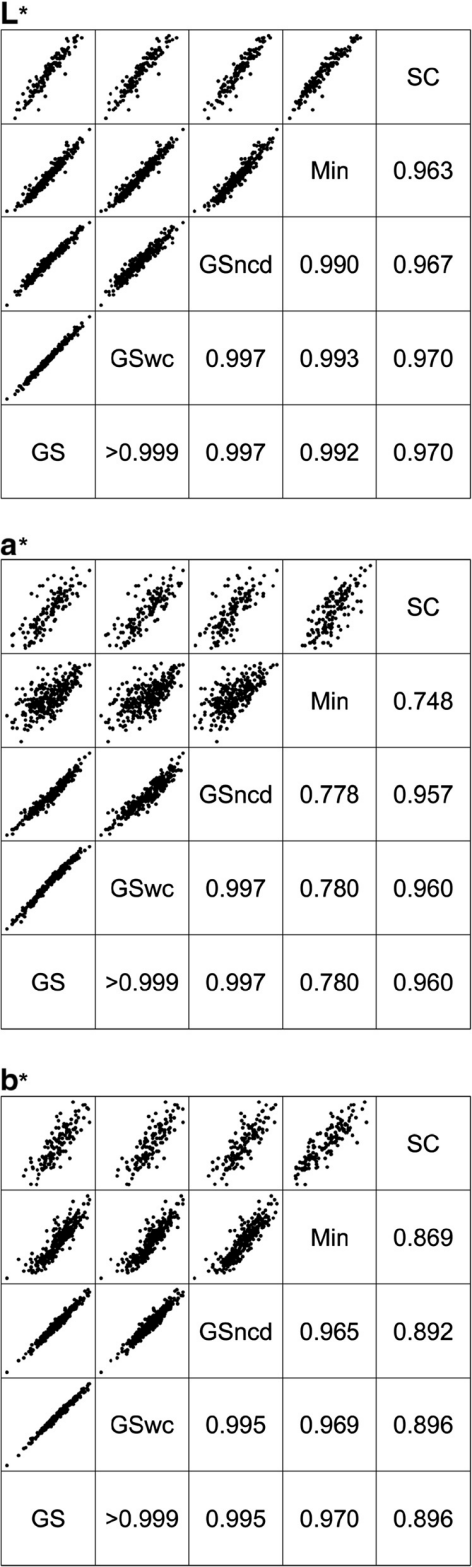
**Correlation of BLUPs for colour traits.** Pairwise plot of BLUPs of packet effects (above the diagonal) and REML estimates of correlations between packet effects (below the diagonal) for colour traits from GrainScan, SmartGrain and SeedCount. Panels represent each colour trait (L*, a* and b*) as labelled. Labels for each method are on the diagonal of each panel: SC (SeedCount), Min (Minolta Colorimeter), GSncd (GrainScan - only those grains where no crease was detected), GSwc (GrainScan – only the non-crease areas of seeds where a crease was detected) and GS (total grain area of all seeds detected by GrainScan).

GrainScan can output colour channel intensity in the standardised CIELAB colourspace. To test whether the crease region on a seed image distorted colour measurements in GrainScan measurements, three ways of calculating colour were tested with GrainScan. Each method measured colour on different parts of the detected seed – the entire seed area (abbreviated GS), the entire seed area of seeds where no crease was detected (abbreviated GSncd) or only the non-crease area of seeds where a crease was detected (abbreviated GSwc). Mean values and ranges (Table 3) agreed very closely between each method, and REML estimates of the correlations between packet effects were all greater than 0.99 (Figure 3). Therefore, for the grain images included in this analysis, the crease area does not effect colour determination, however the option to detect grain crease and differentiate colour measurements based on crease presence is included in the GrainScan interface, a facility that is not available in the other methods considered. While crease detection has only been considered for wheat seeds in this comparison, we anticipate successful detection for any species with a defined crease.

**Table 3:** Summary statistics of raw packet means for colour traits for each method

	**GS**	**GSCD**	**GSNC**	**Minolta**	**SC**
L-min	48.82	49.72	47.36	47.11	43.50
L-mean	57.44	57.67	56.29	51.86	49.78
L-max	66.09	66.27	64.34	58.20	54.80
a*-min	6.25	6.07	6.92	5.50	3.30
a*-mean	9.08	9.00	9.50	6.81	4.74
a*-max	11.46	11.13	12.03	7.94	6.50
b*-min	21.46	21.55	21.95	13.73	15.90
b*-mean	27.69	27.79	27.86	16.89	18.66
b*-max	31.72	31.89	32.18	20.76	21.60

#### Comparison of screening methods

Mean values for colour measurement varied between GrainScan, Minolta and SeedCount (Table 3). REML estimates of correlations between packet effects for colour traits between methods are shown in Figure 3. All methods correlated highly (>0.96) for L* (lightness). GrainScan and SeedCount were strongly correlated for a* (0.96), but less so with Minolta (0.78 and 0.75, respectively). For b*, GrainScan and Minolta were strongly correlated (0.97), compared to SeedCount (0.90 and 0.87 respectively).

Average accuracies (Table 4) were higher for SeedCount (0.988 – 0.995) than GrainScan for all channels (0.874 – 0.988) for both replicated and unreplicated packets. This improved accuracy for colour determination may be due to improved control and uniformity of lighting conditions inside the SeedCount equipment.

**Table 4:** Average accuracies for each colour trait for each method

	**Unreplicated packets**	**Replicated packets**	**Trait**
GrainScan	0.978	0.988	L*
gsCreaseDown	0.979	0.989	
gsNoCrease	0.974	0.986	
SeedCount	0.994	0.995	
GrainScan	0.874	0.930	a*
gsCreaseDown	0.871	0.928	
gsNoCrease	0.867	0.926	
SeedCount	0.992	0.994	
GrainScan	0.926	0.960	b*
gsCreaseDown	0.925	0.960	
gsNoCrease	0.925	0.959	
SeedCount	0.988	0.992	

Averages were computed separately for unreplicated and replicated packets.

Based on these comparisons, GrainScan is an excellent alternative to costly, low throughput methods for standardised colour measurement. GrainScan could be used to determine the presence of genetic variation for colour traits within a population, and where large enough, be sufficiently accurate to conduct complete analysis. Because of its low investment requirement, both in labour and equipment, GrainScan could also be used as an initial investigative tool to determine the value of further investigation with higher cost tools.

#### *Brachypodium distachyon*

**Figure 4:**
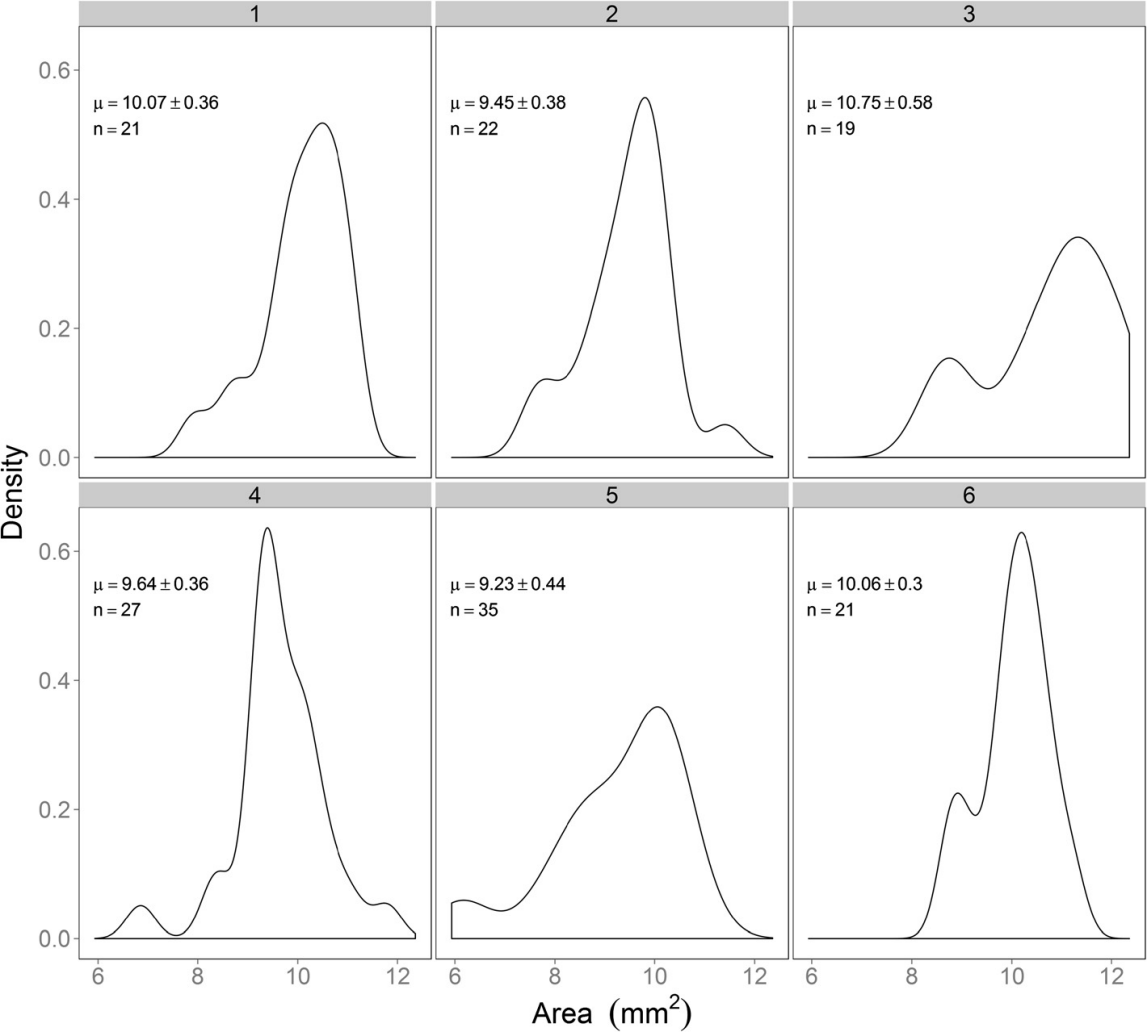
**Density distributions of grain area for six randomly chosen samples of *Brachypodium.*** The mean and confidence interval, along with the number of seeds included in each scan is noted on each panel.

Traits measured for *B.distachyon* seeds were area, perimeter, width and length. Despite the marked difference in shape between seeds from wheat and *B. distachyon*, GrainScan successfully identified seeds, and allowed estimation of mean size as well as variation within a sample (Figure 4, Table 5). The distributions of grain size suggested the possibility of bimodality in these samples, although the sample sizes were much lower than those for wheat. Because of the reduced number of seeds per image, standard errors were higher than those for wheat, highlighting the benefit of scanning larger number of seeds. Since GrainScan can accurately measure seed size across two species with largely differing seed shapes, it is therefore likely that GrainScan can be successfully implemented for many different plant species that also have regular, approximately elliptical morphology.

**Table 5:** **Summary statistics for *B.distachyon *** **size traits**

**Trait**	**Min**	**Mean**	**Max**
Area	7.80	10.00	11.17
Perimeter	20.32	22.94	25.13
Length	7.70	8.71	9.55
Width	1.22	1.47	1.64

## Conclusion

GrainScan enables robust, standardized and detailed study of grain size, shape and colour at very low cost and relatively high throughput. We have demonstrated that size measurements from GrainScan are reproducible between scans, agree well with accepted image analysis techniques, and result in similar rankings of sample material. Because of the dramatically lower cost, and higher throughput of GrainScan compared to other standardized colour measurement methods, GrainScan facilitates detailed study of grain colour in large populations.

GrainScan is freely available as an executable application (http://dx.doi.org/10.4225/08/536302C43FC28).

## Method

### Image capture

Wheat images were scanned using an Epson Perfection V330 (Seiko Epson Corporation, Suwa, Japan) and *B. distachyon* images with a Canon CanoScan LiDE 700 F (Canon Inc, Tokyo, Japan), which are both consumer grade flatbed scanners (<$250 AUD). To standardise image capture, scanning was managed through VueScan (Hamrick Software, http://www.hamrick.com), which allows for a wide range of flatbed scanner manufacturers. All images were scanned at 300 dpi with no colour adjustment or cropping applied. For wheat scanning, grains were spread onto a glass bottomed tray for ease of collection, while for *B. distachyon*, seeds were spread on an overhead transparency film both to avoid scratching the scanner glass and to allow the seeds to be easily collected. Since the wheat seed was bulked from field trial material, a non-uniform subsample of seed was scattered from a seed packet. The operator assessed the appropriate amount of seed to avoid excessive touching of grains. The number of seeds per image ranged from 382 to 985 with a mean value of 654. For *B.distachyon*, seeds were assessed from single spikes from individual plants and all seeds from a spike were measured. The average number of seeds per scan was 18. To maximise contrast at the border of each seed, either a piece of black cardboard, or a matte black box was upturned over the scanning surface, minimizing reflection and shadow. All wheat images used to compare methods are available online [33].

To allow standardisation of colour measurements to the CIELAB colourspace, a Munsell ColorChecker Mini card (X-Rite Corp., MI, USA) was scanned under the same settings as the seed, and used within GrainScan to generate conversion parameters for the colour information measured by the flatbed scanner.

### Image analysis

The image analysis workflow in GrainScan is as follows. A grayscale image is derived from the scanned colour image by averaging the Red and Green channels, since these provide the greatest contrast for seeds considered. Preprocessing is applied to simplify the image prior to segmentation. The functions used in this simplification are mostly connected component (or attribute) morphological operators [34]. These operators are used in preference to older structuring element based morphological functions because they are contour-preserving and there is more selectivity in the way the image is modified. The preprocessing steps include Gaussian smoothing to reduce noise, an attribute closing based on width (0.3 × *Min grain width, a variable accessible to the user*) to fill in the grain crease, a morphological thinning based on elongation to remove any scratches in the background, an attribute opening based on width (0.7 × *Min grain width*) to remove thin debris and an attribute opening based on length (0.7 × *Min grain length*) to remove thick debris.

Because flatbed scanners have uniform lighting and the scanner background provides good contrast with the grain colour, there is no need for sophisticated segmentation techniques. The grains can be separated from the background through simple global thresholding. This threshold is determined using an automated thresholding method, based on a bivariate histogram of input grey level versus gradient, as it is more reliable than methods based on the simple image histogram and is used in image normalisation [35]. Touching grains are separated using a common binary object splitting technique based on finding the troughs between regional maxima in the smoothed distance transform. To remove any small regions created by the grain splitting step, a filtering based on the connected component area (0.5 × *Min grain width* × *Min grain length*) is then performed.

Individual grains are labelled and measurements made of their size and colour. The dimension measurements are area, perimeter, and surrogates for length and width – the major and minor axes of the best fit ellipse (called majellipse and minellipse respectively). These surrogates are quick to compute and tend to be more robust to noise (small bumps and dents) in the segmented grain boundary which can cause problems with algorithms that measure the exact length and width. The dimension units are converted from pixels to millimetres (mm) based on the input *Scanner resolution* in dots per inch (dpi).

The software has two independent options in the analysis of colour. One option is to make the colour measurements for each grain in CIELAB values rather than the raw RGB values measured by the scanner. To use the colour calibration option, the image of a calibrated colour checker card must first be analysed using the ColourCalibration software. This software locates the card, segments each of the colour swatches, extracts the mean RGB values for each swatch, and determines the transformation matrix, RGB2Lab, by linear regression between the measured RGB values and the supplied CIELAB values for each swatch. For convenience, the transformation matrix is saved as two images, one containing the 3×3 matrix and one the 3x1 offset (with filename suffixes of *RGB2Labmat.tif and *RGB2Laboff.tif respectively). By inputting this transformation matrix into the GrainScan software, colour measurements made within each labelled grain can be converted from raw RGB values to calibrated L*, a*, and b* values.

**Figure 5:**
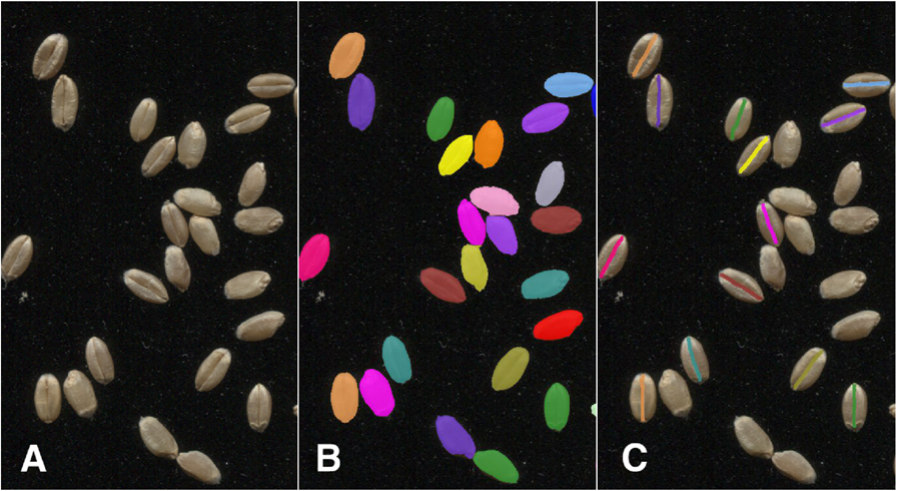
**Examples of GrainScan input and output.** Panel **A**: Scanned wheat grain for GrainScan input. Panel **B**: GrainScan output highlighting segmented grains as determined by the software. Different colours indicate different grains. Panel **C**: Optional crease detection output highlighting regions identified as grain crease.

The second colour analysis option is to detect the grani crease and to make additional colour measurements in the non-crease region and if present, the crease region. The crease detection is performed on each grain by finding the shortest path along the long axis of the grain after mean filtering preferentially along this axis to suppress intensity variability unrelated to the crease.The resulting dimension and colour measurements are saved to a Results sub-directory in Comma Separated Variable (CSV) format. To permit visual inspection of the segmentation results, the labelled grain image and optionally the labelled crease image are saved (with filename suffixes of *.grainLbl.tif and *.creaseLbl.tif respectively). Overlay images with each labelled grain, or crease, overlaid in a different colour on the input image are also saved (with filename suffixes of *.grainOvr.jpg and *.creaseOvr.jpg respectively, Figure 5).

### Comparison to other methods

To compare the image analysis algorithm for size parameters, scanned images were processed with both GrainScan and SmartGrain [29]. Output from these systems was compared to results from a SeedCount system, which was used as a standard for size parameters. SeedCount measurements were taken according to manufacturer’s instructions. To compare between colour measurements determined by GrainScan and SeedCount, output was compared to measurements taken by a Minolta CR-400 chroma meter (Konica Minolta Sensing, Osaka, Japan), an industry standard device for CIE L*, a* and b* values.

### Experimental design

Grain samples were collected from a field trial of a diverse mapping population grown in Leeton, New South Wales. For GrainScan and SmartGrain, seed was scanned from 300 field plots, each of which corresponded to a different genotype. It is important to note that no field replicates of any of the genotypes were available in this study. Prior to scanning, seed was cleaned by a vacuum separator to remove chaff. Packets of seed from each plot were tested using an experimental design in which a proportion (*p* = 0*.*4) of the packets was tested with replication. Thus 120 packets were tested twice and the remaining 180 were tested once. This equated to a total of 420 scans which were conducted by a single operator in 14 batches. Each batch comprised 30 scans done sequentially. Replication was achieved for a packet by tipping out seeds and scanning to obtain the first image, then tipping the seeds back into the packet for a subsequent scan. The second image for any packet was always obtained from a different batch to the first image. Thus the design was a *p −* replicate design [36] with batches as blocks. The SeedCount method was tested on 150 packets, 45 of which were tested with replication, making a total of 195 images. The experimental design was similar to GrainScan and SmartGrain in the sense of involving batches (13 batches with 15 images per batch). Colorimeter (Minolta) measurements were not taken according to a *p*-replicate design with a blocking structure, but were in duplicate for the 300 packets that were included for GrainScan and SmartGrain.

### Data analysis

Analyses were conducted using the ASReml-R package [37] in the R statistical computing environment [38]. For the size data, the analysis commenced with the fitting of a separate mixed model for each trait and method. Since the SeedCount and the SmartGrain methods produce a single value per packet, mean values of the GrainScan data were used to allow comparisons between methods. Each model included random effects for packets and batches. The separate analyses for each method were used to obtain a measure of accuracy for each, defined in terms of the correlation between the predicted packet effects and the true (unknown) packet effects. The data for the different methods were then combined in a multi-variate analysis. The mixed model included a separate mean for each method, random packet effects for each method, random batch effects for each method and a residual for each method. The variance model used for the random packet effects was a factor analytic model [39] which allows for a separate variance for each method and separate correlations between pairs of methods. The other variance models were commensurate with the structure of the experiment. In particular we note that correlations between the GrainScan and SmartGrain methods were included for the batch and residual effects, since these methods were used on the same experimental units (images). The multi-variate analysis provides residual maximum likelihood (REML) estimates of the correlations between the true (unknown) packet effects for different methods. It also provides best linear unbiased predictions (BLUPs) of the packet effects for each method.

For colour measurements, comparisons were made between the complete GrainScan output, GrainScan output for seeds where no crease was detected (abbreviated GSncd), GrainScan output for the non-crease portion of seeds where a crease was detected (abbreviated GSwc), SeedCount and Minolta colorimeter. Since SeedCount and the Minolta methods produce a single value per packet, mean values of the GrainScan data were used to make comparisons between methods.

Initially a separate mixed model analysis was conducted for the data for each trait for each method apart from Minolta. Measurements using the latter were not derived using a design or replication structure as per the other methods and so could not be assessed in the same way. Each model included random effects for packets and batches. The data for the different methods (including Minolta) were then combined in a multivariate analysis. The mixed model was analogous to that used for the seed size analyses.

Brachypodium size analysis was only performed with GrainScan, so no comparisons with other methods were performed.

## Abbreviations

GSncd: GrainScan no crease detected; GSwc: GrainScan with a detected crease; REML: Residual maximum likelihood; BLUP: Best linear unbiased predictor.

## Competing interests

The authors declare that they have no competing interests.

## Authors’ contributions

AW assisted in developing the method, experimental design, conducting the analysis and drafting the manuscript. AS analysed the data and assisted in drafting the manuscript. CC assisted in experimental design, analysis and drafting the manuscript. JR assisted in coordinating the experiment and drafting the manuscript. LS assisted in developing the method, coordinating the experiment and drafting the manuscript. CH assisted in coordinating the experiment and drafting the manuscript. LB developed the image analysis method and assisted in drafting the manuscript. All authors read and approved the final manuscript.
